# Inosine alleviates colorectal cancer liver metastasis by promoting M1 macrophage polarization and modulating the PI3K/AKT signaling pathway

**DOI:** 10.3389/fimmu.2026.1780972

**Published:** 2026-03-11

**Authors:** Yadan Dong, Yuchen Ma, Qun Yu, Zhengmei Shi, Caiyi Wang, Yihao Che, Heng Liu, Desheng Wan, Weizai Shao, Huai Xiao

**Affiliations:** 1 Yunnan Provincial Key Laboratory of Entomological Biopharmaceutical R&D, National-Local Joint Engineering Research Center of Entomoceutics, College of Pharmacy, Dali University, Dali, China; 2 Yunnan Tengyao Pharmaceutical Co., Ltd., Tengchong, China

**Keywords:** colorectal cancer liver metastasis, immunomodulation, inosine, macrophage polarization, PI3K/Akt signaling pathway

## Abstract

**Introduction:**

Colorectal cancer (CRC) represents one of the most common malignancies of the digestive tract, with colorectal cancer liver metastasis (CRLM) constituting a major cause of CRC-related mortality, for which therapeutic options remain limited.

**Methods:**

This study examined the inhibitory role of inosine in CRLM and its molecular mechanisms using *in vitro* and *in vivo* approaches. CRC cell invasion was measured in transwell assays following direct inosine treatment or incubation with conditioned medium from inosine-exposed macrophage co-cultures. A CRLM mouse model generated by intrasplenic MC-38 injection was evaluated for hepatic metastasis. Histopathological examination of liver tissues was performed using HE staining to evaluate metastatic infiltration. Furthermore, flow cytometry and ELISA were utilized to analyze macrophage polarization markers and cytokine expression levels. Through transcriptomic sequencing and analysis, potential target genes and signaling pathways were predicted and subsequently validated by RT-qPCR.

**Results:**

Inosine directly suppressed the invasive abilities of MC-38, CT-26, and HT-29 cells. This inhibitory effect on MC-38 cells was further enhanced under co-culture conditions with M1-polarized macrophages. *In vivo*, inosine reduced the formation of hepatic metastatic nodules, downregulated TNF-α and IL-1β, increased M1 macrophage proportions, and suppressed M2 polarization. Transcriptomics and RT-qPCR indicated that inosine upregulates CYP26A1 and CYP39A1, downregulates BCL-2, PTGS2, and OLFM1, and modulates PI3K/AKT signaling pathway. Inosine inhibits the activation of the PI3K/AKT signaling pathway.

**Discussion:**

Inosine exerted an inhibitory effect on colorectal cancer liver metastasis by skewing macrophages toward an M1 phenotype, dampening pro-inflammatory cytokine release, and regulating key genes via the PI3K/AKT pathway.

## 1 Introduction

Colorectal cancer (CRC) is the second leading cause of cancer-related mortality and one of the most common malignancies of the gastrointestinal system (1). Over the past three decades, the global incidence and mortality rates of CRC have tripled (2), imposing a severe burden on human health and quality of life (3, 4). The high mortality rate of CRC is partly attributed to its elevated metastatic potential (5, 6). Extensive clinical evidence indicates that the liver is the most frequent site of metastasis in advanced CRC, with blood drainage from the gastrointestinal tract via the portal venous system facilitating the dissemination of CRC cells to the liver (7). Colorectal cancer liver metastasis (CRLM) is the most common cause of death in patients with colorectal cancer (8). Therefore, CRLM constitutes a major challenge in the prognosis and treatment of CRC, highlighting an urgent need for novel therapeutic strategies to prevent or inhibit CRC metastasis. Immunotherapies such as programmed cell death-1 (PD 1) and cytotoxic T-lymphocyte-associated protein 4 (CTLA-4) antibodies have revolutionized cancer treatment in multiple cancers; however, they provide modest benefits to patients with CRLM.

The tumor microenvironment (TME) plays a pivotal role in driving cancer progression and determining responses to standard therapies (9). The TME comprises a variety of interacting components, primarily including tumor-associated macrophages (TAMs), CD4^+^ and CD8^+^ T cells, dendritic cells, natural killer cells, tumor-associated endothelial cells, cancer-associated fibroblasts, and myeloid-derived suppressor cells (10, 11). TAMs are among the most abundant immune cells infiltrating the TME, making them a key focus of immunotherapeutic strategies (12, 13). TAMs typically exhibit distinct functional phenotypes. M1-like TAMs exert pro-inflammatory and anti-tumor effects, while M2-like TAMs display anti-inflammatory and pro-tumorigenic functions. M2-like TAMs can promote carcinogenesis, suppress anti-tumor immunity, stimulate angiogenesis, and enhance tumor cell invasion (14, 15). Macrophages are highly plastic immune cells that are critical for maintaining long-term immune homeostasis. They perform essential immune functions such as defense, surveillance, and regulation, serving as key effector cells that modulate immune responses through phagocytosis and cytokine secretion. Macrophages polarize into M1 and M2 phenotypes with distinct roles. M1 macrophages are primarily activated by IFN-γ and LPS (16), characterized by pro-inflammatory and anti-tumor properties and expressing markers such as IL-12, TNF-α, and iNOS. M2 macrophages are mainly induced by IL-4 or IL-13 (17), associated with tissue growth and repair, while exhibiting anti-inflammatory and pro-tumor functions during tumor progression. M2 markers include TGF-β, Arg-1, IL-10, and CD206. Therapeutic strategies targeting TAMs focus on reducing the infiltration of pro-tumorigenic M2-TAMs, reprogramming TAMs toward the anti-tumor M1 phenotype, and activating their phagocytic capacity against tumor cells (18, 19). The M1/M2 TAM ratio has been validated as a prognostic indicator and a potential biomarker for predicting responses to immunotherapy (20).

Inosine (IS) is an endogenous purine nucleoside and a key intermediate in human energy metabolism and nucleic acid synthesis. IS has been proven to improve liver function parameters in patients with chronic hepatitis (21). It exerts regulatory effects on autoimmune hepatitis by protecting hepatocytes and inhibiting excessively activated immune cells (22). Furthermore, IS enhances CD8^+^ T cell proliferation and IFN-γ secretion via activation of the adenosine A2A receptors signaling pathway, promotes tissue-resident memory T cells, and plays a critical role in anti-tumor immunity (23). As an adjuvant in acute/chronic hepatitis and cirrhosis, IS promotes hepatocyte repair, improves abnormal liver function, and reduces the risk of hepatic encephalopathy. Recent studies have confirmed that purine metabolite IS enhances tumor immunogenicity and thus immune checkpoint blockade therapy response by inhibiting the ubiquitin-activating enzyme UBA6 in tumor cells (24). The supplementation with IS enhances the anti-tumor efficacy of immune checkpoint blockade and adoptive T-cell transfer in solid tumors that are defective in metabolizing IS (25). Although IS is already used clinically, its influence on macrophage polarization remains unclear. In the preliminary study of our research group, based on the CT-26 syngeneic transplanted CRC model, IS significantly inhibited tumor proliferation. Moreover, IS increased the proportion of the M1 phenotype among tumor-infiltrating macrophages (26). This study aims to investigate the potential impact of IS on tumor-associated TAMs polarization and CRC metastasis and to explore the mechanisms by which IS modulates macrophage polarization to influence CRC metastasis *in vivo*.

## 2 Materials and methods

### 2.1 Materials

Inosine (I104348) was purchased from Shanghai Aladdin. RPMI-1640 Medium (G4531), DMEM High Glucose Medium (G4511) were purchased from Wuhan Pricella. Fetal Bovine Serum (FBS) was purchased from Cegrogen. Penicillin-Streptomycin Solution (BL505A) was purchased from Hefei Baisha Biotechnology. IFN-γ (061798–1) was purchased from PeproTech. LPS (L8880) was purchased from Beijing Solarbio. APC Rat anti-Mouse CD86 (558703) was purchased from BD Pharmingen. PE Rat anti-Mouse CD206 (2696705) was purchased from Invitrogen. Oxaliplatin (HY-17371) was purchased from MedChemExpress. Mouse TNF-α ELISA Kit (RX202412M), Mouse IL-1β ELISA Kit (RX203063M) were purchased from Quanzhou Ruixin. FITC Rat Anti-Mouse F4/80 (123107) was purchased from BioLegend. Rapid RNA Extraction Kit (AG21023), Evo M-MLV Reverse Transcription Premix Kit (AG11728), SYBR Green Pro Taq HS Premix (AG11701) were purchased from Hunan Accurate. The mouse monocyte-macrophage cell line RAW264.7, mouse colon cancer cell line MC-38, and human normal colon epithelial cell line CCD-841 were purchased from Wuhan Pricella. The mouse colorectal cancer cell line CT-26, human colon cancer cell line HT-29, and human renal proximal tubule epithelial cell line HK-2 were obtained from the Cell Bank of the Chinese Academy of Sciences. Anti-PI3K (WL02240) was purchased from Wanlei Biotech Co., Ltd. Anti-phospho-PI3K (AF3241) and anti-AKT (AF6261)/phospho-AKT (AF0016) were purchased from Affinity Biosciences. Anti-GAPDH (GB15004) was purchased from Wuhan Servicebio.

### 2.2 Cell-based *in vitro* pharmacological assays

#### 2.2.1 Cell culture and polarization induction of macrophages

RAW264.7, HT-29, MC-38, and CCD-841 cells were routinely maintained in DMEM high-glucose medium. CT-26 cells were cultured in RPMI-1640 medium, and HK-2 cells in DMEM/F12 medium. All media were supplemented with 10% FBS and 1% penicillin-streptomycin, and cells were incubated at 37 °C in a humidified atmosphere with 5% CO_2_. To induce polarization, macrophages were treated with 1 µg/mL LPS plus 20 ng/mL IFN-γ for 24 hours to promote M1 polarization.

#### 2.2.2 Co-culture experiment

After polarization, RAW264.7 cells were treated with different concentrations of IS for 24 hours. The conditioned medium was collected and subsequently used to establish a macrophage-colorectal cancer co-culture system with colorectal cancer cells.

#### 2.2.3 Cell viability assay

Cells were plated in 96-well plates at a density of 5 × 10^4^ cells/well and treated with 1.25, 2.5 or 5 mmol/L IS for 24h. For co-cultured MC-38 cells, the conditioned medium collected in section 2.2.2 was used. Then, 3-(4,5-dimethylthiazol-2-yl)-2,5 diphenyl tetrazolium bromide (MTT; 5 mg/mL) was added and the cells were incubated for 3 h. The cell supernatant was removed and replenished with 200 μL of DMSO to dissolve the formazan precipitate, and the optical density was measured at 490 nm using a microplate reader.

#### 2.2.4 Transwell assay

Cells were prepared as a single-cell suspension at 2 × 10^5^ cells/mL in serum-free medium. A 200 µL aliquot of the cell suspension was added to the upper chamber. After 12 hours of incubation at 37°C to allow for polarization and adhesion, the medium was replaced with drug-containing or control medium. For co-cultured MC-38 cells, the conditioned medium collected in section 2.2.2 was used. The cells were further incubated for 48 hours to assess invasion effects. The chambers were then gently washed three times with PBS, fixed with 4% paraformaldehyde for 20 minutes, and stained with 0.1% crystal violet for 30 minutes, followed by rinsing with purified water. Non-specific staining in the upper chamber was mechanically removed with a cotton swab, and the invading cells were counted and recorded under a microscope.

### 2.3 Animal

#### 2.3.1 Experimental models and drug administration

All animal experiments in this study were conducted at Dali University by the guidelines for the care and use of laboratory animals. The Animal Ethics Committee of Dali University evaluated and approved all experimental protocols (2024-pz-034). Forty-five male C57BL/6 mice of SPF grade (18–22 g, License No. SCXK (Beijing) 2024-0001) underwent a 7-day acclimatization period during which they were provided with food and water ad libitum. Mice were randomly divided into six groups: the normal group, the model group, the OXA group, a low-dose IS group (IS-L), a medium-dose IS group (IS-M), and a high-dose IS group (IS-H). A colorectal tumor liver metastasis model was established via intrasplenic injection of tumor cells (27). Six mice were randomly assigned to the normal control group, whereas the remaining animals underwent surgical induction of the experimental model. Under anesthesia, a small incision was made on the left flank to expose the spleen, and approximately 2 × 10^6^ MC-38 cells in 50 µL PBS were injected into the splenic capsule using an insulin syringe with a needle. 24 hours post-inoculation, the normal and model groups received equal volumes of 0.9% NaCl. The oxaliplatin group received 3 mg/kg oxaliplatin, while the IS groups received 25 mg/kg, 50 mg/kg, and 100 mg/kg IS, respectively. Treatments were administered once daily for 16 days.

#### 2.3.2 Body weight and organ indices

The mice were weighed using an electronic balance to measure the body and organ weights. The body weight was recorded from day 0 to day 16. At the end of the experiment, organs including the liver, spleen, lungs, heart, kidneys, and thymus were immediately removed and weighed. The organ index (%) was calculated according to the following formula:


$$\text{organ index }\left(\text{mg}/\text{g}\right)=\frac{\text{organ weight }\left(\text{mg}\right)}{\text{body weight }\left(\text{g}\right)\;}$$

#### 2.3.3 Organ tissue sections

The livers of each group were fixed with formalin, the paraffin-embedded sections were stained with hematoxylin and eosin, and histologic changes were observed under a light microscope.

#### 2.3.4 ELISA assay

At the end of the experiment, blood samples were collected and allowed to stand at room temperature for 1 hour. Serum was separated by centrifugation at 3000 rpm for 10 minutes (4 °C), aspirated, and stored at -80 °C. Cytokine levels of TNF-α and IL-1β were analyzed using ELISA kits according to the manufacturer’s instructions.

#### 2.3.5 Flow cytometry

Mouse spleen tissues were placed in pre-cooled PBS containing 5% FBS and homogenized using a high-speed low-temperature tissue homogenizer. The filtrate was centrifuged at 1200 rpm for 5 minutes at 4 °C, and the supernatant was discarded to retain the cell pellet. Red blood cells were lysed by adding 1 mL of red blood cell lysis buffer, mixing, and incubating for 10 minutes at room temperature in the dark. The lysis was terminated by adding an equal volume of PBS with 3% FBS, followed by two washes under the same centrifugation conditions. Cells were resuspended in PBS with 5% FBS to a density of 1 × 10^7^ cells/mL. Surface antigen staining was performed using F4/80-FITC and CD86-APC antibodies (diluted 1:100 in PBS with 5% FBS) with incubation at 4 °C in the dark for 30 minutes. After fixation and permeabilization at 4 °C for 30 minutes each, intracellular antigen labeling was conducted with CD206-PE antibody (diluted 1:50 in PBS with 5% FBS) and incubated in the dark for 2 hours. All samples were washed twice with PBS containing 3% FBS, filtered to prepare single-cell suspensions, and analyzed by flow cytometry.

#### 2.3.6 Transcriptome analysis of liver tissue

Liver samples from the control, model, and IS-treated groups were collected. Total RNA extraction, library construction, and analysis were performed by Shanghai Personal Biotechnology Co., Ltd. Sequence alignment and gene-specific read counts were obtained using HTSeq (v0.9.1). To ensure comparability of gene expression levels across different genes and samples, expression levels were normalized using the FPKM method. For paired-end sequencing, each fragment generates two reads, and FPKM only counts fragments where both reads align to the same transcript. Differential expression analysis between groups was conducted using DESeq (v1.38.3) with a negative binomial distribution model. Genes with |log_2_FoldChange| > 1 and a *P*-value< 0.05 were considered differentially expressed. KEGG pathway enrichment analysis was performed using clusterProfiler (v4.6.0), with pathways showing a *P*-value< 0.05 considered statistically significant. Gene Set Enrichment Analysis (GSEA, v4.1.0) was employed to examine whether predefined gene sets were enriched at the top or bottom of a ranked list of genes based on their differential expression between two groups, without requiring a strict differential expression threshold.

#### 2.3.7 Quantitative real-time PCR

RNA in the liver tissue was extracted using a Trizol reagent. The extracted RNA was reverse-transcribed into complementary DNA (cDNA) using a commercial kit. Quantitative PCR analyses (10 µL) were conducted according to the ratio of RNase-free water: 2× SYBR Green Pro Taq HS Premix: Forward primer: Reverse primer: cDNA=3.6: 5.0: 0.2: 0.2: 1.0. The expression of genes was calculated using the 2^−ΔΔCt^ method. The gene primers used in this study are listed in **Table 1**.

**Table 1:** The gene-specific primer sequences.

Primer name	Forward primer	Reverse primer
PI3K-mouse	ACACCACGGTTTGGACTATGG	GGCTACAGTAGTGGGCTTGG
AKT-mouse	ATGAACGACGTAGCCATTGTG	TTGTAGCCAATAAAGGTGCCAT
BCL2-mouse	CCGTCGTGACTTCGCAGAGATG	TCCCTGAAGAGTTCCTCCACCAC
CYP26A1-mouse	GCCTCTCCAACCTGCACGATTC	GCTCCAGACAACTGCTGACTTCC
CYP39A1-mouse	ATAGCAATCGCCGTTCTGGGAAG	TCCAGCTCCAATCCAGGGAATCC
OLFM1-mouse	TGGACAGGCGAACTCAGAGAGAC	CCTGGCTAGATGCTGCTTATGGC
PTGS2-mouse	ATGAGTACCGCAAACGCTTCTCC	AGCAGGGCAGGGTACAGTTCC
GAPDH-mouse	GCAAATTCAACGGCACAGTCAAG	TCGCTCCTGGAAGATGGTGATG

#### 2.3.8 Western blot analysis

The total proteins of cells were extracted by RIPA reagent. After boiling at 100 °C for 10 min, the proteins were separated by 10% sodium dodecyl sulfate (SDS)-polyacrylamide gel electrophoresis (PAGE) and transferred to a polyvinylidene difluoride (PVDF) membrane at voltage of 100 V for 1 h. The PVDF membrane was blocked with 5% BSA for 2 h to block non-specific binding at room temperature and incubated with primary antibodies at 4 °C overnight. The membranes were rinsed with TBST for 3 times to wash away the unconjugated primary antibodies. After incubation with horseradish peroxidase (HRP) conjugated secondary antibodies at room temperature for 2 h. The protein immune-complexes were prepared with enhanced chemiluminescence (ECL).

### 2.4 Statistical analysis

All data were analyzed using SPSS 23.0 software. Comparisons between two groups were made using a t-test, while one-way ANOVA was applied for comparisons among multiple groups. *P* < 0.05 indicated a statistically significant difference. Measurement data were presented as mean ± standard error of mean (SEM).

## 3 Results

### 3.1 Viability of MC-38 cells

**Figure 1:**
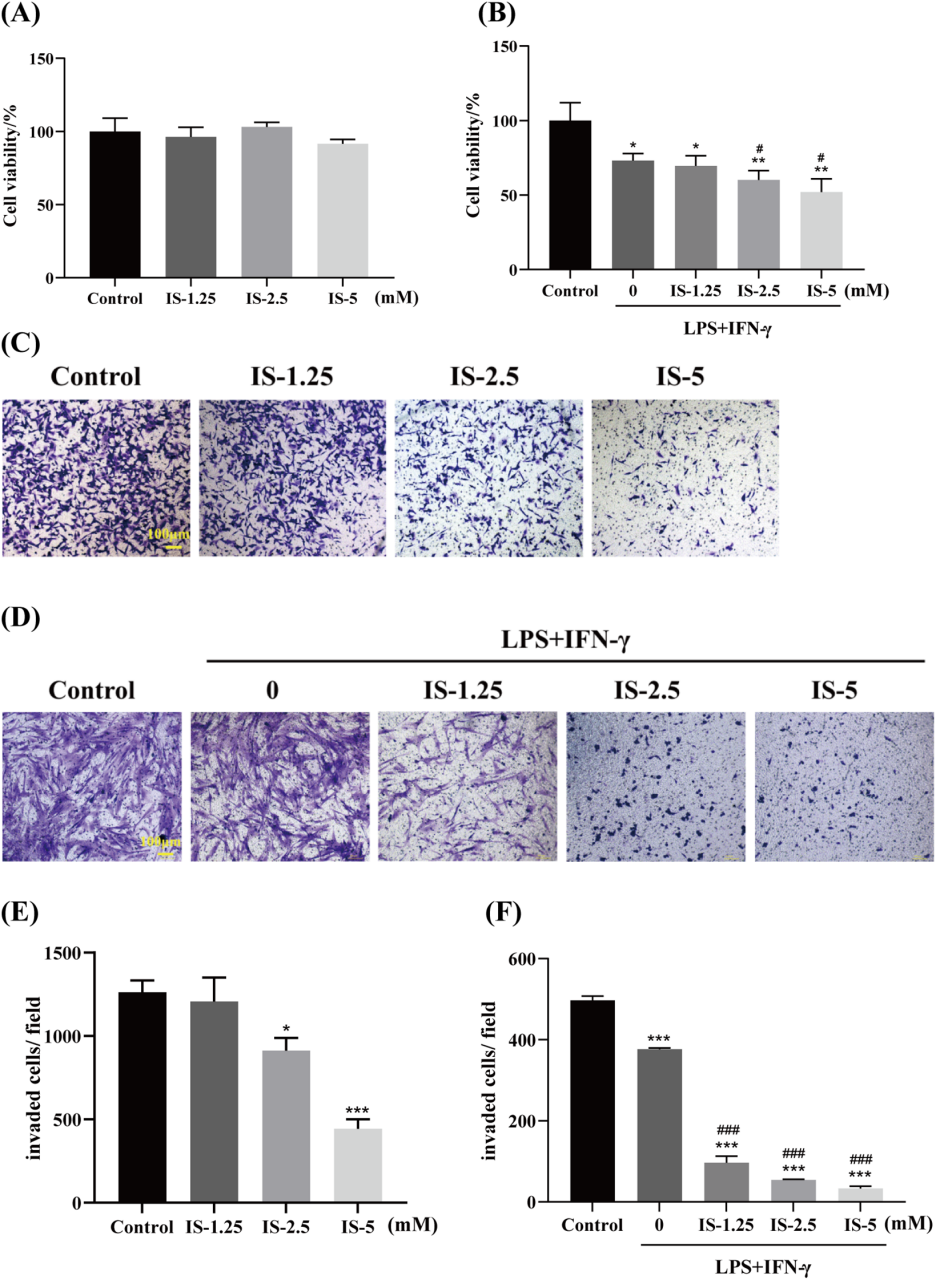
Effects of IS on the proliferation and invasion of MC−38 cells (n=3). **(A, B)** Viability of MC−38 cells after direct IS and indirect IS administration via co−culture. **(C, D)** Representative images of MC−38 cells on the outer side of the transwell membrane after direct IS treatment and indirect IS treatment via co−culture. **(E, F)** Quantitative analysis of invaded MC−38 cell numbers after direct IS treatment and indirect IS treatment via co−culture. Compared to the control group: ^*^P < 0.05, ^**^P < 0.01, ^***^P < 0.001; Compared with the LPS+IFN-γ induced M1 group: ^#^*P* < 0.05, ^###^*P* < 0.001.

No significant difference in cell viability was found between each group with direct IS administration and the control group (*P* ≥ 0.05), which indicated that IS had no obvious inhibitory effect on the proliferation of MC38 cells via direct administration (**Figure 1A**). The cell viability of all co-culture groups was markedly reduced compared with the control group. In contrast to the IS-untreated co-culture group, IS treatment under the co-culture condition further suppressed the viability of MC38 cells, with significant differences observed at the concentrations of 2.5 mM and 5 mM (*P* < 0.05) (**Figure 1B**). These results indicate that the inhibitory effect of IS on the proliferation of colorectal cancer cells may be related to the promotion of M1 macrophage polarization.

### 3.2 Effects of IS on the invasive ability of MC-38 cells

Transwell assay results demonstrated a significant reduction in the number of invasive cells in medium-dose and high−dose IS−treated groups compared to the control group (*P* < 0.05 or *P* < 0.001) (**Figures 1C, E**). These findings indicated that IS could directly inhibit the invasive ability of MC-38 cells. MC-38 cells were co-cultured with a conditioned medium of M1 macrophages (with or without IS) for 24 hours. The conditioned medium of M1 macrophages without IS could inhibit the number of invasive cells (*P* < 0.001). The conditioned medium of M1 macrophages with IS could further and significantly suppressed the invasive capacity of MC-38 cells (*P* < 0.001) (**Figures 1D, F**), showing a dose-dependent response and a stronger inhibitory effect than direct IS administration alone. The results indicated that the inhibitory effect of IS on the invasion of colorectal cancer cells may be associated with the promotion of M1 macrophage polarization.

### 3.3 Effects of IS on cellular invasive capacity

**Figure 2:**
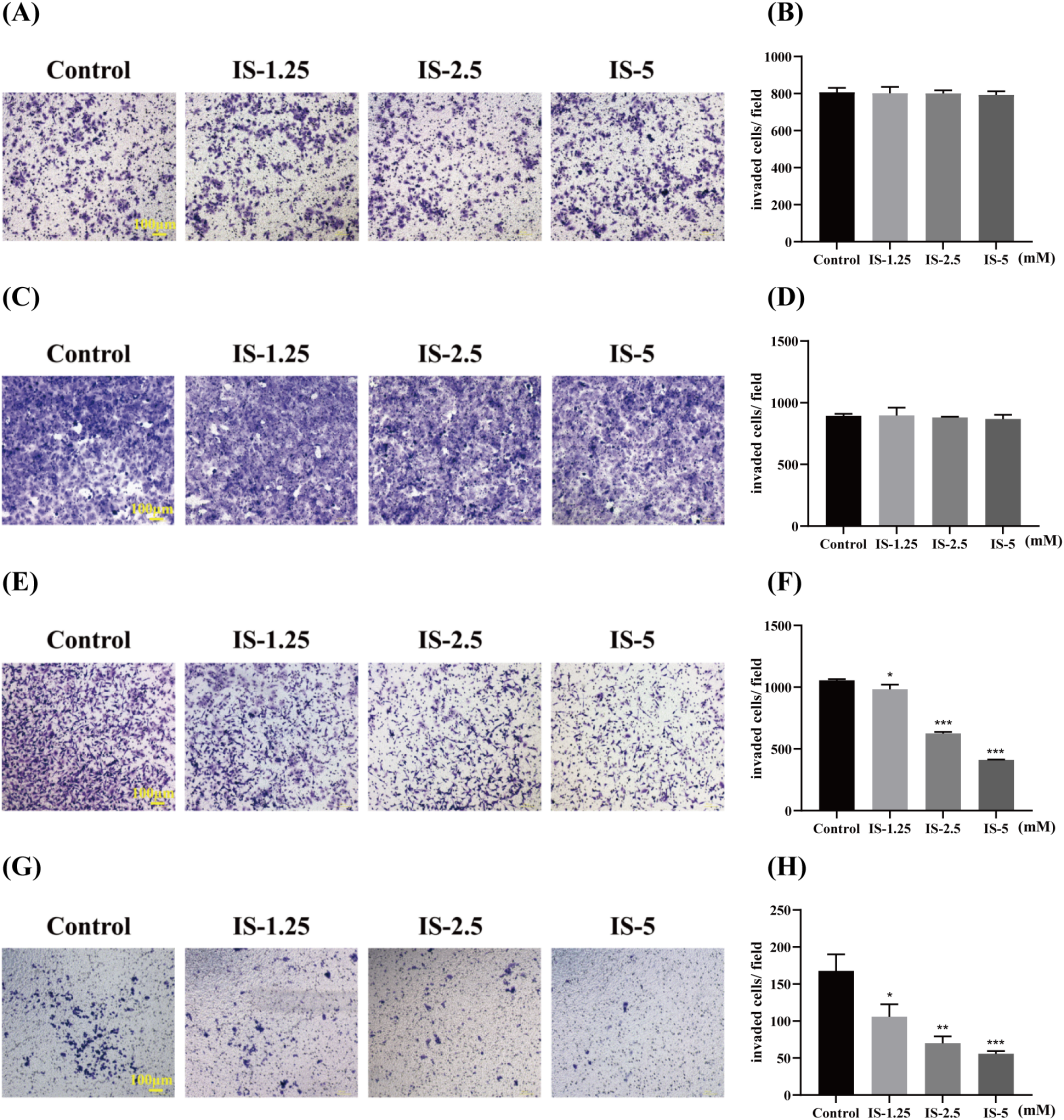
Effects of IS on cell invasion (n = 3). **(A, C, E, G)** Representative images of CCD-841, HK-2, CT-26, and HT-29 cells on the outer side of the transwell membrane following direct IS treatment, respectively. **(B, D, F, H)** Quantitative analysis of the number of invaded CCD-841, HK-2, CT-26, and HT-29 cells after direct IS treatment, respectively. Compared to the control group: ^*^P < 0.05, ^**^P < 0.01, ^***^P < 0.001.

To more comprehensively evaluate the effect of inosine on colorectal cancer liver metastasis, Transwell assays were performed using multiple cell lines. MC-38 and CT-26 are mouse colorectal cancer cell lines with high invasive and liver metastatic potential. HT29, a human colorectal cancer cell line, was used to verify the findings in a human cell background and enhance the clinical relevance of the study. CCD-841 normal colon epithelial cells and HK-2 human renal tubular epithelial cells served as normal controls to confirm whether inosine exerts a selective inhibitory effect on tumor cells. Compared to the control group, no significant difference in the number of invasive cells was observed in any of the IS treatment groups (*P* ≥ 0.05) (**Figures 2A–D**). The number of invasive cells in the IS-treated groups was significantly reduced compared to the control group (*P* < 0.05) (**Figures 2E–H**), exhibiting a dose-dependent effect. These results indicated that IS did not significantly inhibit the invasive capacity of normal cells like CCD-841 and HK-2 cells, but could directly inhibit the invasive ability of cancer cells like CT-26 and HT-29 cells.

### 3.4 Mouse body weight and organ indices

**Figure 3:**
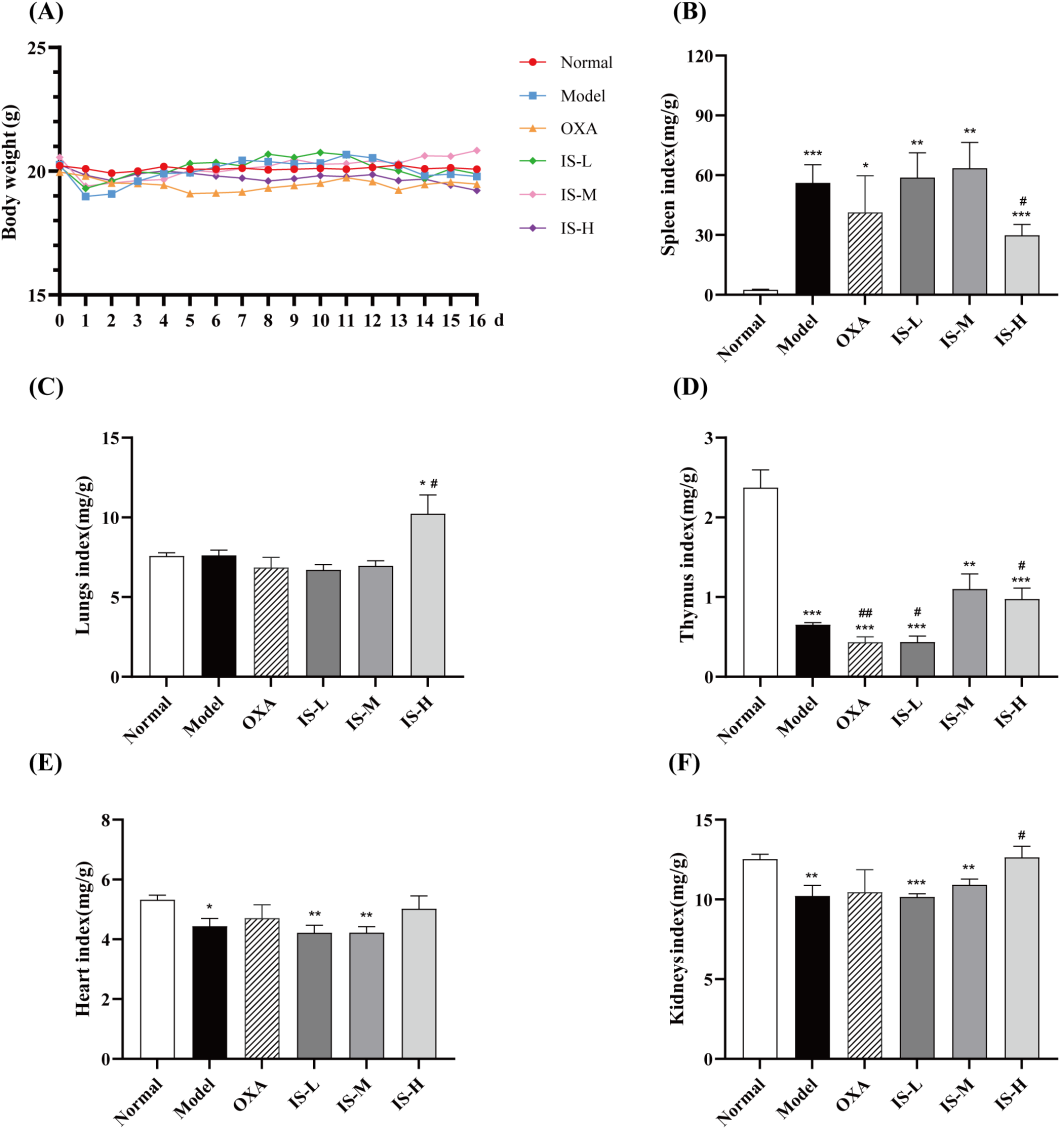
**(A)** Effect of IS on mouse body weight. **(B-F)** Organ indices of the spleen, lungs, thymus, heart, and kidneys, respectively. Normal and model groups: n = 6; IS-L and IS-M groups: n = 7; OXA and IS-H groups: n = 5. Compared to the normal group: ^*^P < 0.05, ^**^P < 0.01, ^***^P < 0.001; Compared to the model group: ^#^*P* < 0.05, ^##^*P* < 0.01.

The changes in mouse body weight are shown in **Figure 3A**. Compared with the normal group, no significant differences in body weight changes were observed in the model group and the IS treatment groups. In contrast, the body weight of mice in the oxaliplatin group began to decline gradually from day 3. Deaths occurred starting on day 12 of administration, with an overall mortality rate of 20.0%. Specifically, one mouse died in each of the low-dose and medium-dose IS groups, two mice died in both the model group and the high-dose IS group, and three mice died in the oxaliplatin group. Postmortem examination revealed that the deceased mice had developed severe tumor progression. The death of mice in the IS-treated group was mainly related to tumor burden. The death of mice in the oxaliplatin group may be caused by drug toxicity, and it is speculated that the mouse death might be attributed to the impaired liver metabolic function after modeling. As shown in **Figures 3B–F** compared with the normal group, the spleens of all model mice developed large tumor masses, and the spleen index of the model group increased significantly (*P* < 0.001). Compared with the model group, high-dose IS significantly reduced the spleen index (*P* < 0.05), indicating that IS not only inhibits metastatic tumors but also effectively suppresses the growth of primary tumors, with a superior inhibitory effect compared to oxaliplatin. IS exerts a certain protective effect on the kidneys and heart of mice. Compared with the model group, the medium- and high-dose IS groups significantly restored the thymus index, suggesting that IS may treat CRLM by enhancing immune function and modulating thymic activity to rebuild adaptive immune responses.

### 3.5 Status of hepatic metastases and histopathological observation by HE staining

**Figure 4:**
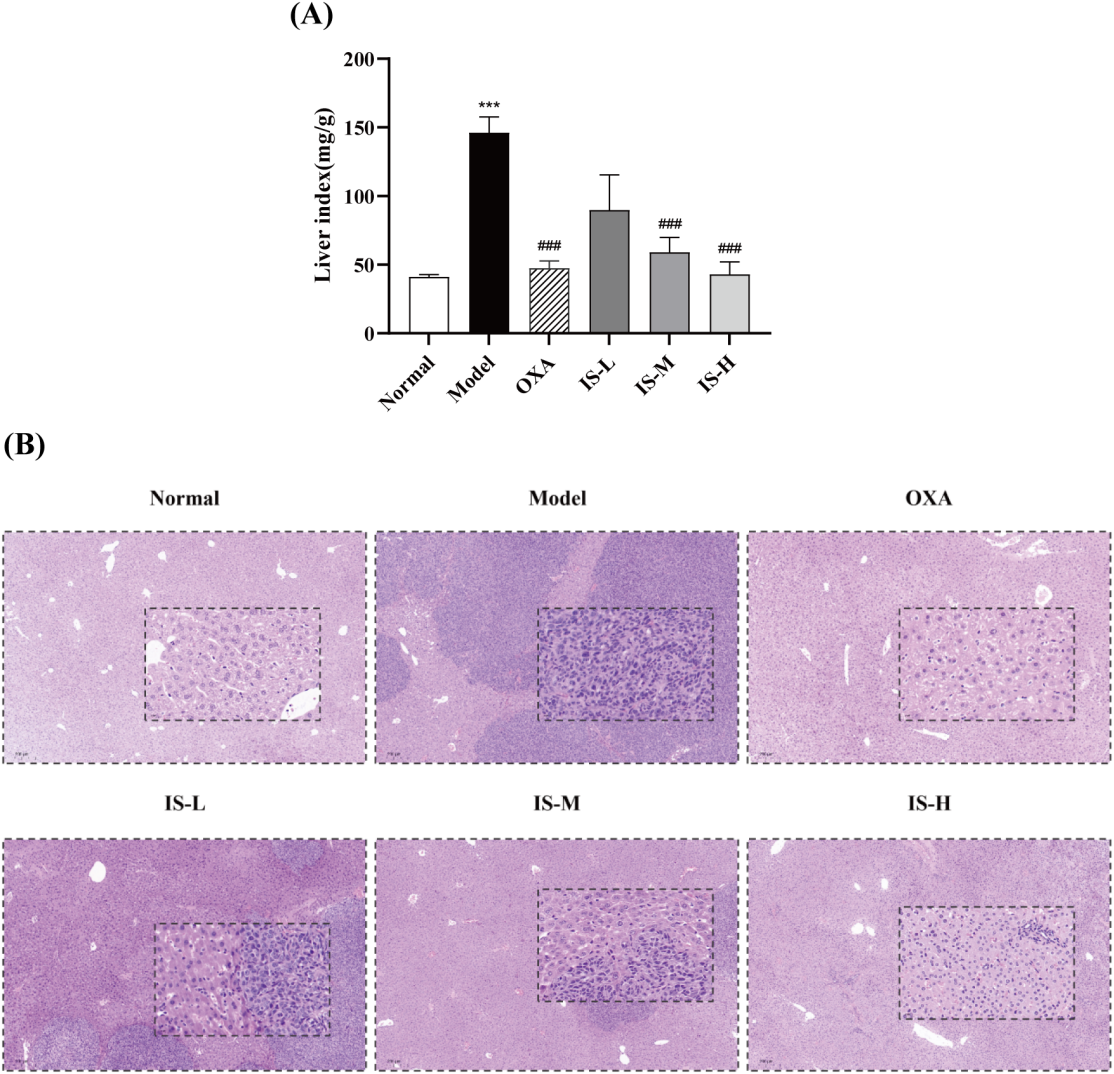
**(A)** Effect of IS on the liver index in mice with CRLM. Normal and model groups: n = 6; IS-L and IS-M groups: n = 7; OXA and IS-H groups: n = 5. **(B)** HE-stained sections of mouse liver tumor tissues (5×, 20×). Compared to the normal group: ***P < 0.001; Compared to the model group: ^###^*P* < 0.001.

In the model group, hepatic metastatic tumors appeared as white masses distributed extensively across the liver surface with fused growth patterns. In contrast, metastases in the oxaliplatin and IS treatment groups were predominantly located at the liver margins and were markedly reduced compared to the model group(**Supplementary Figure 1**). Liver indices were significantly decreased following treatment with IS or oxaliplatin (*P* < 0.001) (**Figure 4A**), indicating that IS treatment could ameliorate colorectal cancer liver metastasis. **Figure 4B** displays representative histopathological features. Normal hepatocytes exhibited low nuclear-to-cytoplasmic ratios, orderly arrangement, absence of fibrous tissue hyperplasia or inflammatory infiltration, and clear boundaries. In contrast, metastatic tumor cells showed enlarged nuclei, high nuclear-to-cytoplasmic ratios, disordered arrangement, invasive growth, and indistinct boundaries. The liver tissue in the model group was largely invaded by tumor cells. Compared to the model group, both oxaliplatin and IS treatment groups significantly reduced tumor cell metastasis.

### 3.6 Effects of IS on cytokine levels in serum of CRLM mice

**Figure 5:**
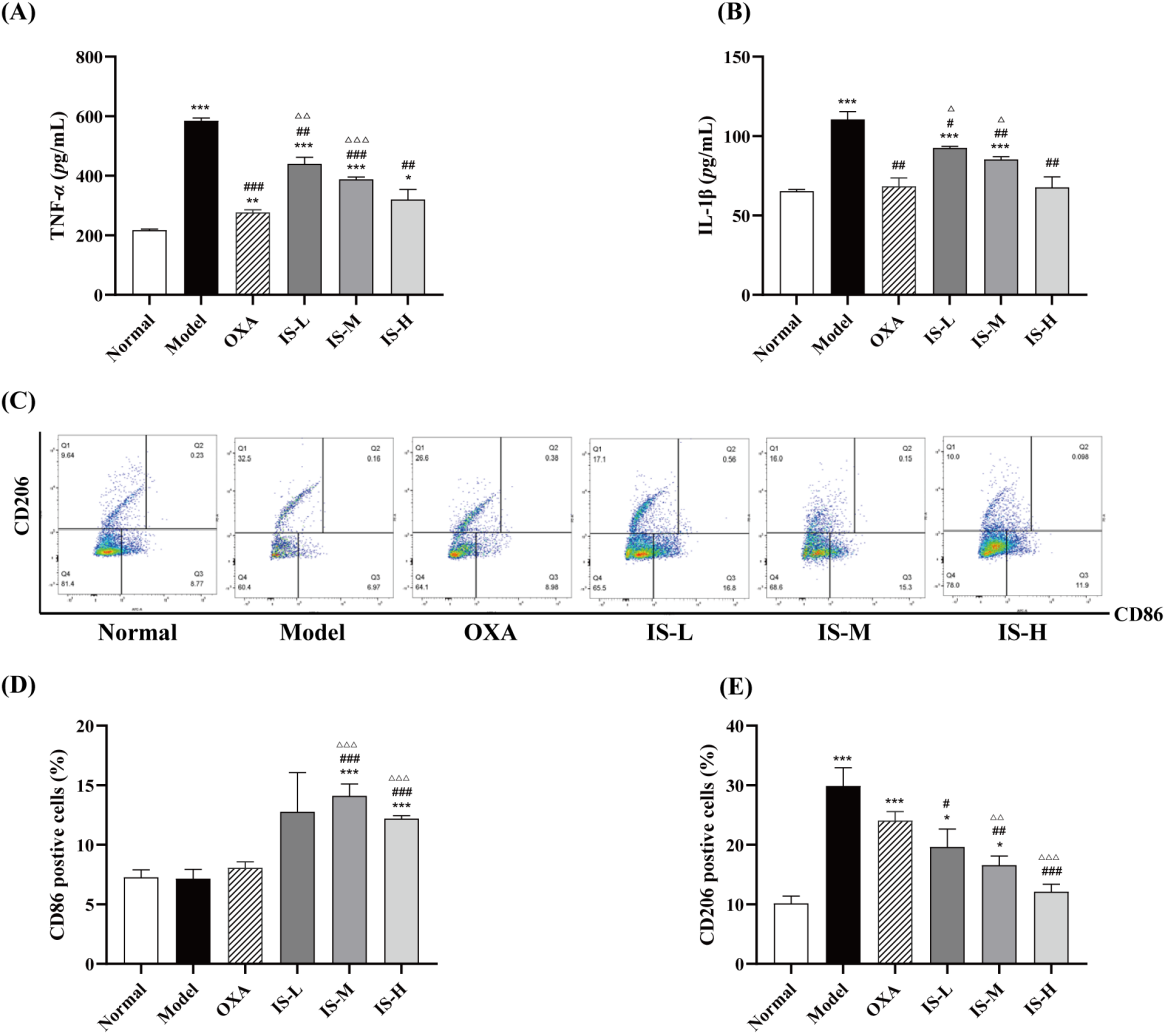
**(A, B)** Quantitative analysis of serum TNF-α and IL-1β levels in CRLM mice treated with IS (n = 3). **(C)** Representative CD86/CD206 dual-fluorescence staining dot plots of macrophages in each group; Q1: M2 (CD86^-^CD206^+^), Q3: M1 (CD86^+^CD206^-^). **(D, E)** Statistical results of the proportions of CD86^+^ and CD206^+^ cells in splenocytes, respectively (n = 3). Compared to the normal group: ^*^P < 0.05, ^**^P < 0.01, ^***^P < 0.001; Compared to the model group: ^#^*P* < 0.05, ^##^*P* < 0.01, ^###^*P* < 0.001; Compared to the OXA group: ^△^*P* < 0.05, ^△△^*P* < 0.01, ^△△△^*P* < 0.001.

Compared with the normal group, serum levels of TNF-α and IL-1β were significantly elevated in the model group (*P* < 0.05). In contrast, oxaliplatin treatment markedly reduced these cytokine levels, restoring them to near-normal values. All doses of IS significantly attenuated the upregulation of TNF-α and IL-1β in the serum of model mice (*P* < 0.05), exhibiting a dose-dependent effect. Notably, administration of IS at 100 mg/kg restored cytokine levels to those comparable to the normal group (**Figures 5A, B**). These results suggest that IS may effectively alleviate the inflammatory response in CRLM by downregulating the expression of the pro-inflammatory cytokines TNF-α and IL-1β.

### 3.7 The proportions of CD86 and CD206 cells in the spleens of CRLM mice

Compared with the normal group, the proportion of cells positive for the M2 phenotype marker CD206 increased significantly in the model group (*P* < 0.001), while the proportion positive for the M1 phenotype marker CD86 showed no significant change, consistent with the macrophage phenotype distribution typically observed in the tumor microenvironment. Oxaliplatin treatment did not significantly alter the proportions of CD86 or CD206 cells compared with the model group, indicating that oxaliplatin does not affect macrophage phenotype ratios in the tumor microenvironment. In contrast, IS treatment significantly increased the proportion of CD86 cells (*P* < 0.001), consistent with previous *in vitro* findings, and significantly decreased the proportion of CD206 cells (*P* < 0.001) in a dose-dependent manner (**Figures 5C–E**). The results indicated that IS may further inhibit tumor progression and metastasis by shifting macrophage polarization from the pro-tumor M2 phenotype to the anti-tumor M1 phenotype.

### 3.8 Mechanism exploration of IS inhibition on CRLM based on transcriptomic sequencing

**Figure 6:**
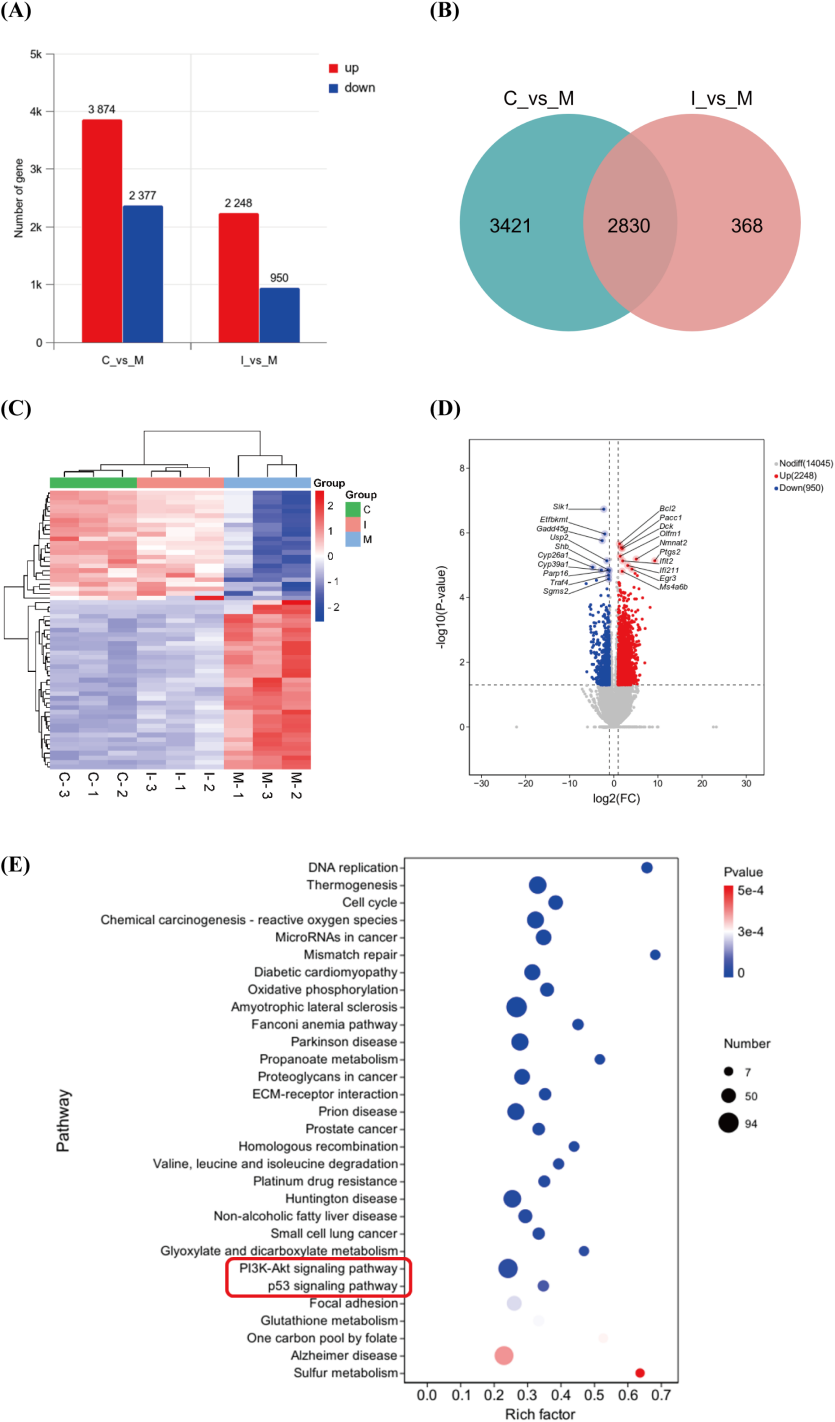
**(A)** DEGs between the normal and model groups, and between the model and IS-treated groups. **(B)** Venn diagram illustrating common DEGs between the comparisons of normal vs. model groups and model vs. IS-treated groups. **(C)** Hierarchical clustering analysis of DEG expression levels across the normal, model, and IS-treated groups. Each group consists of three biological replicates. **(D)** Volcano plot of DEGs between the model and IS-treated groups (top 10). **(E)** KEGG enrichment analysis of DEGs between the model and IS-treated groups. C: normal group; M: model group; I: IS-H group; *P* < 0.05, |log_2_FC| > 1.

Transcriptomic sequencing was performed on liver tissues from mice in the normal group, CRLM model group, and high-dose IS treatment group (n = 3). DEGs were screened using HTSeq for statistical comparison (*P* < 0.05, |log_2_Fold Change| > 1). A total of 6251 DEGs were identified between the normal and model groups, while 3198 DEGs were identified between the model and IS treatment groups (**Figure 6A**). The intersection of these two sets yielded 2830 overlapping DEGs. From these, 61 significantly differentially expressed genes were identified (**Figure 6B**). The hierarchical clustering heatmap based on these 61 genes (**Figure 6C**), demonstrated high intra-group consistency among samples from the normal, model, and IS intervention groups, with distinct separation of gene expression patterns. The volcano plot in **Figure 6D** highlighted 20 core differentially expressed genes (10 upregulated and 10 downregulated). Five genes were selected for further validation due to their relevance. Cytochrome P450 family 26 subfamily A member 1 (CYP26A1) (28, 29), cytochrome P450 family 39 subfamily A member 1 (CYP39A1) (30), B-cell lymphoma 2 (BCL-2) (31–33) and prostaglandin-endoperoxide synthase 2 (PTGS2) (34, 35) were associated with immune-related tumor microenvironment while olfactomedin 1 (OLFM1) (36) were associated with the development and metastasis of colorectal cancer. Based on P−values, among the top 30 enriched pathways, the PI3K/AKT and p53 signaling pathways exhibited significant enrichment alongside disease pathways such as amyotrophic lateral sclerosis (**Figure 6E**). As shown in **Table 2**, considering the number of enriched DEGs, the PI3K/AKT signaling pathway demonstrated stronger relevance. The PI3K/AKT signaling axis serves as a central hub regulating malignant phenotypes of tumor cells, activating downstream effectors through phosphorylation cascades to drive cell proliferation, apoptosis resistance, and metabolic reprogramming. Its aberrant activation has been reported in various malignancies, including ovarian cancer (37), gastric cancer (38), and breast cancer (39).

**Table 2:** Top 20 KEGG pathways of differentially expressed genes between the model group and the IS group.

Pathway ID	Pathway	level1	level2	Up	Down	DEG
mmu05014	Amyotrophic lateral sclerosis	Human Diseases	Neurodegenerative disease	39	55	94
mmu04151	PI3K–Akt signaling pathway	Environmental Information Processing	Signal transduction	76	10	86
mmu05010	Alzheimer disease	Human Diseases	Neurodegenerative disease	27	57	84
mmu05016	Huntington disease	Human Diseases	Neurodegenerative disease	22	51	73
mmu04714	Thermogenesis	Organismal Systems	Environmental adaptation	25	46	71
mmu05012	Parkinson disease	Human Diseases	Neurodegenerative disease	18	51	69
mmu05208	Chemical carcinogenesis–reactive oxygen	Human Diseases	Cancer: overview	11	56	67
mmu05020	Prion disease	Human Diseases	Neurodegenerative disease	22	45	67
mmu05415	Diabetic cardiomyopathy	Human Diseases	Cardiovascular disease	18	42	60
mmu05205	Proteoglycans in cancer	Human Diseases	Cancer: overview	49	8	57
mmu05206	MicroRNAs in cancer	Human Diseases	Cancer: overview	51	5	56
mmu04510	Focal adhesion	Cellular Processes	Cellular community - eukaryotes	50	2	52
mmu04110	Cell cycle	Cellular Processes	Cell growth and	46	2	48
mmu04932	Non-alcoholic fatty liver disease	Human Diseases	Endocrine and metabolic disease	6	38	44
mmu00190	Oxidative phosphorylation	Metabolism	Energy metabolism	0	43	43
mmu05215	Prostate cancer	Human Diseases	Cancer: specific	27	6	33
mmu04512	ECM-receptor interaction	Environmental Information Processing	Signaling molecules and interaction	27	4	31
mmu05222	Small cell lung cancer	Human Diseases	Cancer: specific	26	5	31
mmu01524	Platinum drug resistance	Human Diseases	Drug resistance: antineoplastic	17	11	28
mmu04115	p53 signaling pathway	Cellular Processes	Cell growth and	20	5	25

### 3.9 Mechanistic validation

**Figure 7:**
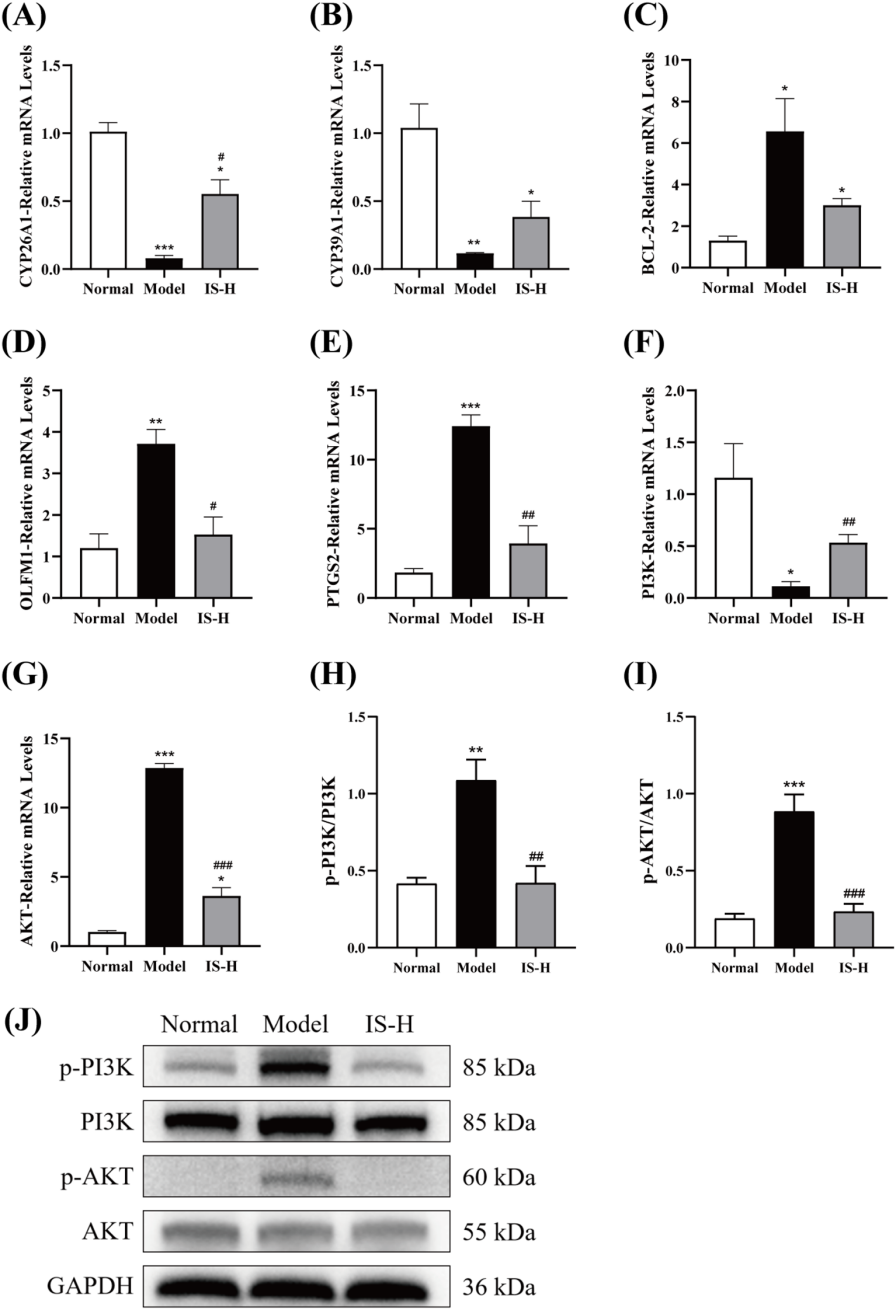
**(A–E)** Effect of IS on the mRNA expression levels of selected differentially expressed genes CYP26A1, CYP39A1, BCL-2, OLFM1 and PTGS2 in the liver of CRLM mice (n=3). **(F, G)** Effect of IS on the mRNA expression levels of PI3K and AKT in the liver of CRLM mice (n=3). **(H-J)** Western blot assay was performed to detect the level of p-PI3K, PI3K, p-AKT, and AKT (n=3). Compared to the normal group: ^*^P < 0.05, ^**^P < 0.01, ^***^P < 0.001; Compared to the model group: ^#^*P* < 0.05, ^##^*P* < 0.01, ^###^*P* < 0.001.

RT-qPCR results revealed that compared to the control group, the expression levels of CYP26A1 and CYP39A1 in the model group were significantly downregulated (*P* < 0.01), while the expression levels of BCL-2, OLFM1, and PTGS2 were significantly upregulated (*P* < 0.05). After treatment with IS, compared to the model group, the expression level of CYP26A1 in the IS-treated group was significantly restored (*P* < 0.05), and CYP39A1 expression also showed an increasing trend. In contrast, the expression levels of OLFM1 and PTGS2 were significantly suppressed (*P* < 0.05), while BCL-2 expression exhibited a decreasing trend (**Figures 7A–E**). Compared to the control group, the expression level of PI3K in the model group was significantly downregulated (*P* < 0.05), while AKT expression was significantly upregulated (*P* < 0.001). Following IS treatment, compared to the model group, the expression level of PI3K in the IS-treated group was significantly restored (*P* < 0.01), and AKT expression was significantly inhibited (*P* < 0.001) (**Figures 7F, G**). Compared with the control group, the levels of p-PI3K and p-AKT in the model group were significantly increased (*P* < 0.01). Compared with the model group, the expressions of p-PI3K and p-AKT in the IS-H group were significantly decreased (*P* < 0.01) (**Figures 7H, I**). The results demonstrated that IS may further inhibit the anti-apoptotic effect of BCL2 and the pro-inflammatory mediator release function of PTGS2 by suppressing the PI3K/AKT signaling pathway, leading to a reduction in the levels of the inflammatory factors TNF-α and IL-1β. IS may maintain the homeostasis of all-trans retinoic acid (ATRA) and cholesterol metabolism by upregulating the expression of CYP26A1 and CYP39A1, respectively, thereby ensuring the normal differentiation and metabolic functions of cells. In addition, it may regulate cell adhesion and signal transduction by downregulating the expression of OLFM1, thereby inhibiting the proliferation and invasion of tumor cells.

## 4 Discussion

Studies have shown that promoting M1 macrophage polarization can inhibit cancer development and metastasis (40). Our preliminary research demonstrated that IS promoted M1 polarization of RAW264.7 cells. Co-culture of CT-26 cells with IS-supplemented M1 macrophage-conditioned medium further potentiated this polarization effect, leading to more significant inhibition of CT-26 cell proliferation and migration. In the present study, co-culture of MC-38 cells with IS-supplemented M1 macrophage-conditioned medium and transwell assay results revealed that IS not only directly inhibited the invasive ability of MC-38 cells but also further enhanced this inhibitory effect under M1 macrophage co-culture conditions. These findings indicated that IS could directly inhibit tumor cell invasion while also indirectly suppressing tumor invasion by modulating macrophage polarization toward the M1 phenotype. This suggested that IS exerted its effects not only through immunomodulation but also via direct intervention in tumor cell biology to inhibit metastasis. IS had no significant effect on the invasive ability of normal CCD-841 and HK-2 cells, whereas it markedly inhibited the invasion of MC-38, CT-26, and HT-29 cells. This demonstrated the tumor cell-specificity of IS, providing a safety basis for its potential clinical application.

Colorectal cancer liver metastasis is one of the leading causes of death in CRC patients. We injected MC-38 cells into the splenic capsule of C57 mice to simulate the process of CRC metastasis to the liver via the portal venous system. The results showed that IS significantly inhibited the growth of liver metastatic tumors, with efficacy comparable to the oxaliplatin. This finding was consistent with the *in vitro* results demonstrating IS’s inhibition of tumor cell invasion, further validating its potential in suppressing tumor metastasis. Oxaliplatin reduced cytokine levels and tumor burden without affecting CD86/CD206 ratios, suggesting macrophage-independent mechanisms. Oxaliplatin exerts its anti-tumor effects mainly by directly killing tumor cells, inhibiting angiogenesis, and regulating T cell-mediated adaptive immunity. Additionally, H&E staining results indicated that IS significantly reduced metastatic nodules in the liver, suggesting that it may inhibit tumor cell invasion and colonization.

M1-polarized macrophages recruit new Th1 cells through chemokines CXCL9 and CXCL10 and produce pro-inflammatory cytokines such as TNF-α, IL-1β, IL-6, IL-12, and IL-23 (41). TNF-α, as a pleiotropic cytokine, exerts biological effects that depend on the integration of microenvironmental signals. While it can promote tumor progression under certain conditions, it may also contribute to the formation of an immunosuppressive microenvironment, thereby exhibiting pro-tumor effects. Clinical studies have shown that TNF-α levels in the ascites of ovarian cancer patients are positively correlated with the extent of metastasis (42). IL-1β, a representative multifunctional cytokine, drives tumor cell proliferation, epithelial-mesenchymal transition, and angiogenesis. Clinicopathological analyses have demonstrated that IL-1β is significantly overexpressed in melanoma, colorectal cancer, and non-small cell lung cancer and is associated with reduced overall survival in patients (43). In this study, IS treatment significantly reduced serum levels of TNF-α and IL-1β in mice, suggesting that IS may inhibit tumor invasion and metastasis by attenuating inflammatory responses. Furthermore, IS significantly increased the proportion of M1 macrophages while decreasing the proportion of M2 macrophages. This result aligned with our previous *in vitro* findings that IS promotes M1 polarization, further supporting the hypothesis that IS exerts anti-tumor effects by modulating macrophage phenotypes.

Combined transcriptomic sequencing and KEGG enrichment analysis revealed five target genes (CYP26A1, CYP39A1, BCL2, OLFM1, and PTGS2) implicated in the effects of IS, and demonstrated that the PI3K/AKT signaling pathway played a critical role in IS-mediated regulation of macrophage polarization. CYP26A1 is a critical metabolic enzyme primarily responsible for the degradation of retinoic acid (RA), which plays an important role in regulating immune cell function. Overexpression of CYP26A1 may reduce RA levels in the tumor microenvironment, thereby suppressing anti-tumor immune responses and promoting immune evasion (44). CYP39A1, an enzyme involved in cholesterol metabolism, is mainly responsible for the metabolism of oxysterols (45). It may influence tumor cell proliferation, invasion, and metastasis by modulating cholesterol levels, thereby participating in tumor cell metabolic reprogramming. Cholesterol metabolites, such as oxysterols, play significant roles in regulating immune cell function. CYP39A1 may affect immune responses in the tumor microenvironment by altering oxysterol levels. Overexpression of BCL-2 enhances tumor cell survival by inhibiting apoptosis, thereby promoting tumor growth and metastasis (46). OLFM1 is upregulated in various tumors and may promote tumor cell proliferation and invasion by regulating cell adhesion and signal transduction (36). It may also influence anti-tumor immune responses by modulating the function of immune cells in the tumor microenvironment. PTGS2 (also known as COX-2) is a key enzyme in prostaglandin synthesis, involved in inflammatory responses and tumorigenesis (47). CYP26A1 expression was significantly restored in the IS-treated group, and CYP39A1 expression showed an upward trend. In contrast, OLFM1 and PTGS2 expression levels were significantly suppressed, while BCL-2 expression exhibited a downward trend. Although transcriptomic analysis identified five relevant differentially expressed genes, their specific functions in the action of IS require further validation through gene knockout or overexpression experiments.

Numerous studies have shown that the progression and metastasis of colorectal cancer are often mediated through the PI3K/AKT pathway (48). The PI3K/AKT pathway is a crucial signaling cascade regulating cell proliferation, survival, and metabolism. This pathway involves growth factors and receptor tyrosine kinases (RTKs), which are co-activated (49). RTK activation triggers PI3K activation, generating PIP3 and subsequently activating AKT kinase (50). AKT activation leads to the phosphorylation and regulation of various downstream targets, including transcription factors, cell cycle regulators, and components of the mTOR pathway. Recent studies have also linked PI3K/AKT pathway activation to macrophage polarization. PI3K inhibition can enhance NF-κB activation and iNOS production, thereby increasing M1 macrophage polarization markers. Conversely, PI3K inhibition negatively affects metastasis-related factors and stimulates the expression of M2 macrophage surface markers (51, 52). RT-qPCR validation results showed that PI3K expression was significantly restored in the IS-treated group, while AKT expression was significantly inhibited. The discrepancy in trends may be attributed to the fact that PI3K activity primarily depends on post-translational modifications, and mRNA expression levels may not reflect actual protein activity. The results of Western blot analysis showed that the protein expression levels of p-PI3K and p-AKT were significantly decreased in the IS treatment group, indicating that IS may improve the progression of CRLM by inhibiting the activation of the PI3K/AKT signaling pathway. Moreover, the precise mechanism by which IS regulates the PI3K/AKT pathway to influence macrophage polarization remains to be elucidated through molecular biology experiments or rescue experiments using PI3K inhibitors. Future studies may explore the combined effects of IS with chemotherapy drugs or immune checkpoint inhibitors to evaluate its potential in comprehensive cancer therapy.

## 5 Conclusion

IS may inhibit the proliferation and invasion abilities of colorectal cancer cells by promoting the polarization of macrophages toward the M1 phenotype. IS can directly inhibit tumor cell invasion without exerting obvious toxicity on normal cells. Furthermore, it improves CRLM in mice by promoting the infiltration of M1-like TAMs and attenuating the activation of the PI3K/AKT signaling pathway. This study provided crucial experimental evidence for the clinical application of IS, while also offering novel insights for the development of immunomodulation-based anti-tumor therapeutic strategies.

## Data Availability

The data presented in the study are deposited in the National Center for Biotechnology Information (NCBI) repository, accession number PRJNA1433715.

## References

[1] Bray F Laversanne M Sung H Ferlay J Siegel RL Soerjomataram I . Global cancer statistics 2022: GLOBOCAN estimates of incidence and mortality worldwide for 36 cancers in 185 countries. CA Cancer J Clin. (2024) 74:229–63. doi: 10.3322/caac.21834, PMID: 38572751

[2] GBD 2019 Colorectal Cancer Collaborators . Global, regional, and national burden of colorectal cancer and its risk factors, 1990-2019: a systematic analysis for the Global Burden of Disease Study 2019. Lancet Gastroenterol Hepatol. (2022) 7:627–47. doi: 10.1016/s2468-1253(22)00044-9, PMID: 35397795 PMC9192760

[3] Gupta S . Screening for colorectal cancer. Hematol Oncol Clin North Am. (2022) 36:393–414. doi: 10.1016/j.hoc.2022.02.001, PMID: 35501176 PMC9167799

[4] Siegel RL Wagle NS Cercek A Smith RA Jemal A . Colorectal cancer statistics, 2023. CA Cancer J Clin. (2023) 73:233–54. doi: 10.3322/caac.21772, PMID: 36856579

[5] Siegel RL Miller KD Fedewa SA Ahnen DJ Meester RGS Barzi A . Colorectal cancer statistics, 2017. CA Cancer J Clin. (2017) 67:177–93. doi: 10.3322/caac.21395, PMID: 28248415

[6] van der Geest LG Lam-Boer J Koopman M Verhoef C Elferink MA de Wilt JH . Nationwide trends in incidence, treatment and survival of colorectal cancer patients with synchronous metastases. Clin Exp Metastasis. (2015) 32:457–65. doi: 10.1007/s10585-015-9719-0, PMID: 25899064

[7] Kow AWC . Hepatic metastasis from colorectal cancer. J Gastrointest Oncol. (2019) 10:1274–98. doi: 10.21037/jgo.2019.08.06, PMID: 31949948 PMC6955002

[8] Wu Y Yang S Ma J Chen Z Song G Rao D . Spatiotemporal immune landscape of colorectal cancer liver metastasis at single-cell level. Cancer Discov. (2022) 12:134–53. doi: 10.1158/2159-8290.Cd-21-0316, PMID: 34417225

[9] Hiam-Galvez KJ Allen BM Spitzer MH . Systemic immunity in cancer. Nat Rev Cancer. (2021) 21:345–59. doi: 10.1038/s41568-021-00347-z, PMID: 33837297 PMC8034277

[10] Mao X Xu J Wang W Liang C Hua J Liu J . Crosstalk between cancer-associated fibroblasts and immune cells in the tumor microenvironment: new findings and future perspectives. Mol Cancer. (2021) 20:131. doi: 10.1186/s12943-021-01428-1, PMID: 34635121 PMC8504100

[11] Zhang Q Wang J Yadav DK Bai X Liang T . Glucose metabolism: the metabolic signature of tumor associated macrophage. Front Immunol. (2021) 12:702580. doi: 10.3389/fimmu.2021.702580, PMID: 34267763 PMC8276123

[12] Asgharzadeh S Salo JA Ji L Oberthuer A Fischer M Berthold F . Clinical significance of tumor-associated inflammatory cells in metastatic neuroblastoma. J Clin Oncol. (2012) 30:3525–32. doi: 10.1200/jco.2011.40.9169, PMID: 22927533 PMC3675667

[13] Seton-Rogers S . Taming TAMs in brain metastases. Nat Rev Cancer. (2022) 22:2–3. doi: 10.1038/s41568-021-00426-1, PMID: 34799680

[14] Sunakawa Y Stintzing S Cao S Heinemann V Cremolini C Falcone A . Variations in genes regulating tumor-associated macrophages (TAMs) to predict outcomes of bevacizumab-based treatment in patients with metastatic colorectal cancer: results from TRIBE and FIRE3 trials. Ann Oncol. (2015) 26:2450–6. doi: 10.1093/annonc/mdv474, PMID: 26416897 PMC4658546

[15] Xiang X Wang J Lu D Xu X . Targeting tumor-associated macrophages to synergize tumor immunotherapy. Signal Transduct Target Ther. (2021) 6:75. doi: 10.1038/s41392-021-00484-9, PMID: 33619259 PMC7900181

[16] Yang X Cai S Shu Y Deng X Zhang Y He N . Exosomal miR-487a derived from m2 macrophage promotes the progression of gastric cancer. Cell Cycle. (2021) 20:434–44. doi: 10.1080/15384101.2021.1878326, PMID: 33522393 PMC7894454

[17] Seong JB Kim B Kim S Kim MH Park YH Lee Y . Macrophage peroxiredoxin 5 deficiency promotes lung cancer progression via ROS-dependent M2-like polarization. Free Radic Biol Med. (2021) 176:322–34. doi: 10.1016/j.freeradbiomed.2021.10.010, PMID: 34637923

[18] Georgoudaki AM Prokopec KE Boura VF Hellqvist E Sohn S Östling J . Reprogramming tumor-associated macrophages by antibody targeting inhibits cancer progression and metastasis. Cell Rep. (2016) 15:2000–11. doi: 10.1016/j.celrep.2016.04.084, PMID: 27210762

[19] Majeti R Chao MP Alizadeh AA Pang WW Jaiswal S Gibbs KD Jr. . CD47 is an adverse prognostic factor and therapeutic antibody target on human acute myeloid leukemia stem cells. Cell. (2009) 138:286–99. doi: 10.1016/j.cell.2009.05.045, PMID: 19632179 PMC2726837

[20] Shanmugam MK Lee JH Chai EZ Kanchi MM Kar S Arfuso F . Cancer prevention and therapy through the modulation of transcription factors by bioactive natural compounds. Semin Cancer Biol. (2016) 40-41:35–47. doi: 10.1016/j.semcancer.2016.03.005, PMID: 27038646

[21] Klysz DD Fowler C Malipatlolla M Stuani L Freitas KA Chen Y . Inosine induces stemness features in CAR-T cells and enhances potency. Cancer Cell. (2024) 42:266–282.e8. doi: 10.1016/j.ccell.2024.01.002, PMID: 38278150 PMC10923096

[22] Yang F Zhou L Shen Y Zhao S Zheng Y Men R . Metabolic heterogeneity caused by HLA-DRB1*04:05 and protective effect of inosine on autoimmune hepatitis. Front Immunol. (2022) 13:982186. doi: 10.3389/fimmu.2022.982186, PMID: 35990653 PMC9389112

[23] Mager LF Burkhard R Pett N Cooke NCA Brown K Ramay H . Microbiome-derived inosine modulates response to checkpoint inhibitor immunotherapy. Science. (2020) 369:1481–9. doi: 10.1126/science.abc3421, PMID: 32792462

[24] Wang T Gnanaprakasam JNR Chen X Kang S Xu X Sun H . Inosine is an alternative carbon source for CD8(+)-T-cell function under glucose restriction. Nat Metab. (2020) 2:635–47. doi: 10.1038/s42255-020-0219-4, PMID: 32694789 PMC7371628

[25] Zhang L Jiang L Yu L Li Q Tian X He J . Inhibition of UBA6 by inosine augments tumour immunogenicity and responses. Nat Commun. (2022) 13:5413. doi: 10.1038/s41467-022-33116-z, PMID: 36109526 PMC9478149

[26] Ma Y Qian X Yu Q Dong Y Wang J Liu H . Inosine prevents colorectal cancer progression by inducing M1 phenotypic polarization of macrophages. Molecules. (2024) 30:123. doi: 10.3390/molecules30010123, PMID: 39795180 PMC11721193

[27] Yue L Xu X Dai S Xu F Zhao W Gu J . Orosomucoid 1 promotes colorectal cancer progression and liver metastasis by affecting PI3K/AKT pathway and inducing macrophage M2 polarization. Sci Rep. (2023) 13:14092. doi: 10.1038/s41598-023-40404-1, PMID: 37640741 PMC10462626

[28] Bhattacharya N Yuan R Prestwood TR Penny HL DiMaio MA Reticker-Flynn NE . Normalizing microbiota-induced retinoic acid deficiency stimulates protective CD8(+) T cell-mediated immunity in colorectal cancer. Immunity. (2016) 45:641–55. doi: 10.1016/j.immuni.2016.08.008, PMID: 27590114 PMC5132405

[29] Wang RC Liu ZK Chen W Yang Y Peng JP . The antitumor immunopreventive effects of a DNA vaccine against CYP26a1 on mouse breast carcinoma. Vaccine. (2011) 29:8915–23. doi: 10.1016/j.vaccine.2011.09.066, PMID: 21959331

[30] Kim KH Park YL Park SY Joo YE . Expression of an oxysterol-metabolizing enzyme in colorectal cancer and its relation to tumor cell behavior and prognosis. Pathol Res Pract. (2023) 251:154875. doi: 10.1016/j.prp.2023.154875, PMID: 37820439

[31] Kaloni D Diepstraten ST Strasser A Kelly GL . BCL-2 protein family: attractive targets for cancer therapy. Apoptosis. (2023) 28:20–38. doi: 10.1007/s10495-022-01780-7, PMID: 36342579 PMC9950219

[32] Luo F Li H Ma W Cao J Chen Q Lu F . The BCL-2 inhibitor APG-2575 resets tumor-associated macrophages toward the M1 phenotype, promoting a favorable response to anti-PD-1 therapy via NLRP3 activation. Cell Mol Immunol. (2024) 21:60–79. doi: 10.1038/s41423-023-01112-y, PMID: 38062129 PMC10757718

[33] Zhao L Liu P Mao M Zhang S Bigenwald C Dutertre CA . BCL2 inhibition reveals a dendritic cell-specific immune checkpoint that controls tumor immunosurveillance. Cancer Discov. (2023) 13:2448–69. doi: 10.1158/2159-8290.Cd-22-1338, PMID: 37623817 PMC7615270

[34] Hashemi Goradel N Najafi M Salehi E Farhood B Mortezaee K . Cyclooxygenase-2 in cancer: A review. J Cell Physiol. (2019) 234:5683–99. doi: 10.1002/jcp.27411, PMID: 30341914

[35] Venè R Costa D Augugliaro R Carlone S Scabini S Casoni Pattacini G . Evaluation of glycosylated PTGS2 in colorectal cancer for NSAIDS-based adjuvant therapy. Cells. (2020) 9:683. doi: 10.3390/cells9030683, PMID: 32168749 PMC7140631

[36] Shi W Ye Z Zhuang L Li Y Shuai W Zuo Z . Olfactomedin 1 negatively regulates NF-κB signalling and suppresses the growth and metastasis of colorectal cancer cells. J Pathol. (2016) 240:352–65. doi: 10.1002/path.4784, PMID: 27555280

[37] Ediriweera MK Tennekoon KH Samarakoon SR . Role of the PI3K/AKT/mTOR signaling pathway in ovarian cancer: Biological and therapeutic significance. Semin Cancer Biol. (2019) 59:147–60. doi: 10.1016/j.semcancer.2019.05.012, PMID: 31128298

[38] Fattahi S Amjadi-Moheb F Tabaripour R Ashrafi GH Akhavan-Niaki H . PI3K/AKT/mTOR signaling in gastric cancer: Epigenetics and beyond. Life Sci. (2020) 262:118513. doi: 10.1016/j.lfs.2020.118513, PMID: 33011222

[39] Guerrero-Zotano A Mayer IA Arteaga CL . PI3K/AKT/mTOR: role in breast cancer progression, drug resistance, and treatment. Cancer Metastasis Rev. (2016) 35:515–24. doi: 10.1007/s10555-016-9637-x, PMID: 27896521

[40] Ho TTB Nasti A Seki A Komura T Inui H Kozaka T . Combination of gemcitabine and anti-PD-1 antibody enhances the anticancer effect of M1 macrophages and the Th1 response in a murine model of pancreatic cancer liver metastasis. J Immunother Cancer. (2020) 8:e001367. doi: 10.1136/jitc-2020-001367, PMID: 33188035 PMC7668383

[41] Genard G Lucas S Michiels C . Reprogramming of tumor-associated macrophages with anticancer therapies: radiotherapy versus chemo- and immunotherapies. Front Immunol. (2017) 8:828. doi: 10.3389/fimmu.2017.00828, PMID: 28769933 PMC5509958

[42] Balkwill F . TNF-alpha in promotion and progression of cancer. Cancer Metastasis Rev. (2006) 25:409–16. doi: 10.1007/s10555-006-9005-3, PMID: 16951987

[43] Kaneko N Kurata M Yamamoto T Morikawa S Masumoto J . The role of interleukin-1 in general pathology. Inflammation Regen. (2019) 39:12. doi: 10.1186/s41232-019-0101-5, PMID: 31182982 PMC6551897

[44] Zhu Y Zhou T Zheng Y Yao Y Lin M Zeng C . Folate metabolism-associated CYP26A1 is a clinico-immune target in colorectal cancer. Genes Immun. (2025) 26:376–93. doi: 10.1038/s41435-025-00342-6, PMID: 40604317 PMC12353796

[45] Li D Yu T Hu J Wu J Feng S Xu Q . Downregulation of CYP39A1 serves as a novel biomarker in hepatocellular carcinoma with worse clinical outcome. Oxid Med Cell Longev. (2021) 2021:5175581. doi: 10.1155/2021/5175581, PMID: 35003516 PMC8741352

[46] Thor Straten P Andersen MH . The anti-apoptotic members of the Bcl-2 family are attractive tumor-associated antigens. Oncotarget. (2010) 1:239–45. doi: 10.18632/oncotarget.134, PMID: 21304176 PMC3248102

[47] Zhu Q Han Y He Y Meng P Fu Y Yang H . Quercetin inhibits neuronal Ferroptosis and promotes immune response by targeting lipid metabolism-related gene PTGS2 to alleviate breast cancer-related depression. Phytomedicine. (2024) 130:155560. doi: 10.1016/j.phymed.2024.155560, PMID: 38815404

[48] Fresno Vara JA Casado E de Castro J Cejas P Belda-Iniesta C González-Barón M . PI3K/Akt signalling pathway and cancer. Cancer Treat Rev. (2004) 30:193–204. doi: 10.1016/j.ctrv.2003.07.007, PMID: 15023437

[49] Mahajan K Mahajan NP . PI3K-independent AKT activation in cancers: a treasure trove for novel therapeutics. J Cell Physiol. (2012) 227:3178–84. doi: 10.1002/jcp.24065, PMID: 22307544 PMC3358464

[50] Kong D Yamori T . Advances in development of phosphatidylinositol 3-kinase inhibitors. Curr Med Chem. (2009) 16:2839–54. doi: 10.2174/092986709788803222, PMID: 19689267

[51] Xia W Hou M . Macrophage migration inhibitory factor rescues mesenchymal stem cells from doxorubicin-induced senescence though the PI3K-Akt signaling pathway. Int J Mol Med. (2018) 41:1127–37. doi: 10.3892/ijmm.2017.3282, PMID: 29207187

[52] Zhao SJ Kong FQ Jie J Li Q Liu H Xu AD . Macrophage MSR1 promotes BMSC osteogenic differentiation and M2-like polarization by activating PI3K/AKT/GSK3β/β-catenin pathway. Theranostics. (2020) 10:17–35. doi: 10.7150/thno.36930, PMID: 31903103 PMC6929615

