# Supplementary material for: Inosine alleviates colorectal cancer liver metastasis by promoting M1 macrophage polarization and modulating the PI3K/AKT signaling pathway
Source: Front Immunol. 2026 Mar 11;17:1780972. doi: 10.3389/fimmu.2026.1780972 (PMC13013416; doi:10.3389/fimmu.2026.1780972)
Supplement: Supplementary file 1 [file DataSheet1.docx]

Supplementary Material

# Supplementary Figures


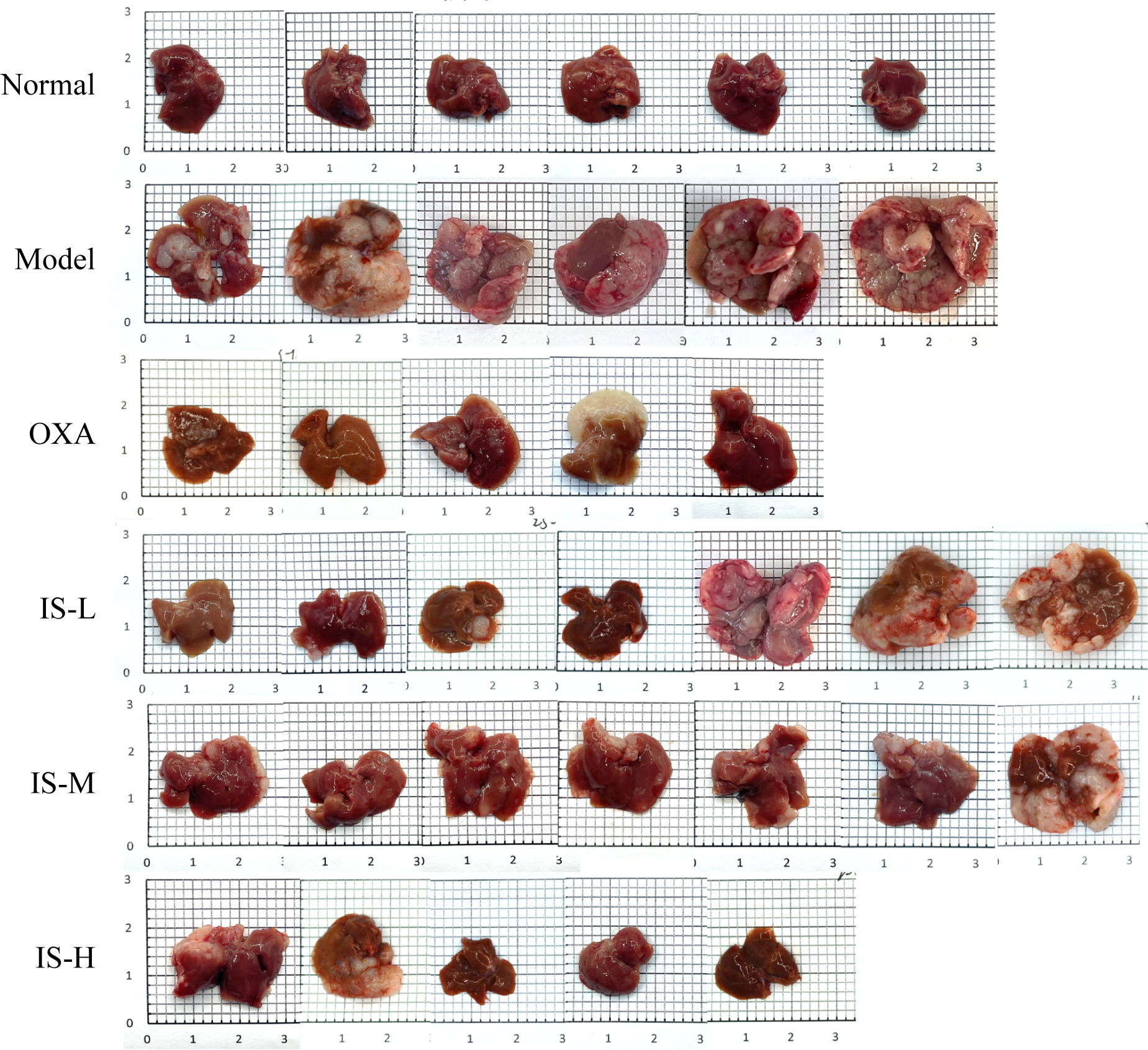


**Supplementary Figure 1.** Gross morphology of liver tumor tissues in mice.


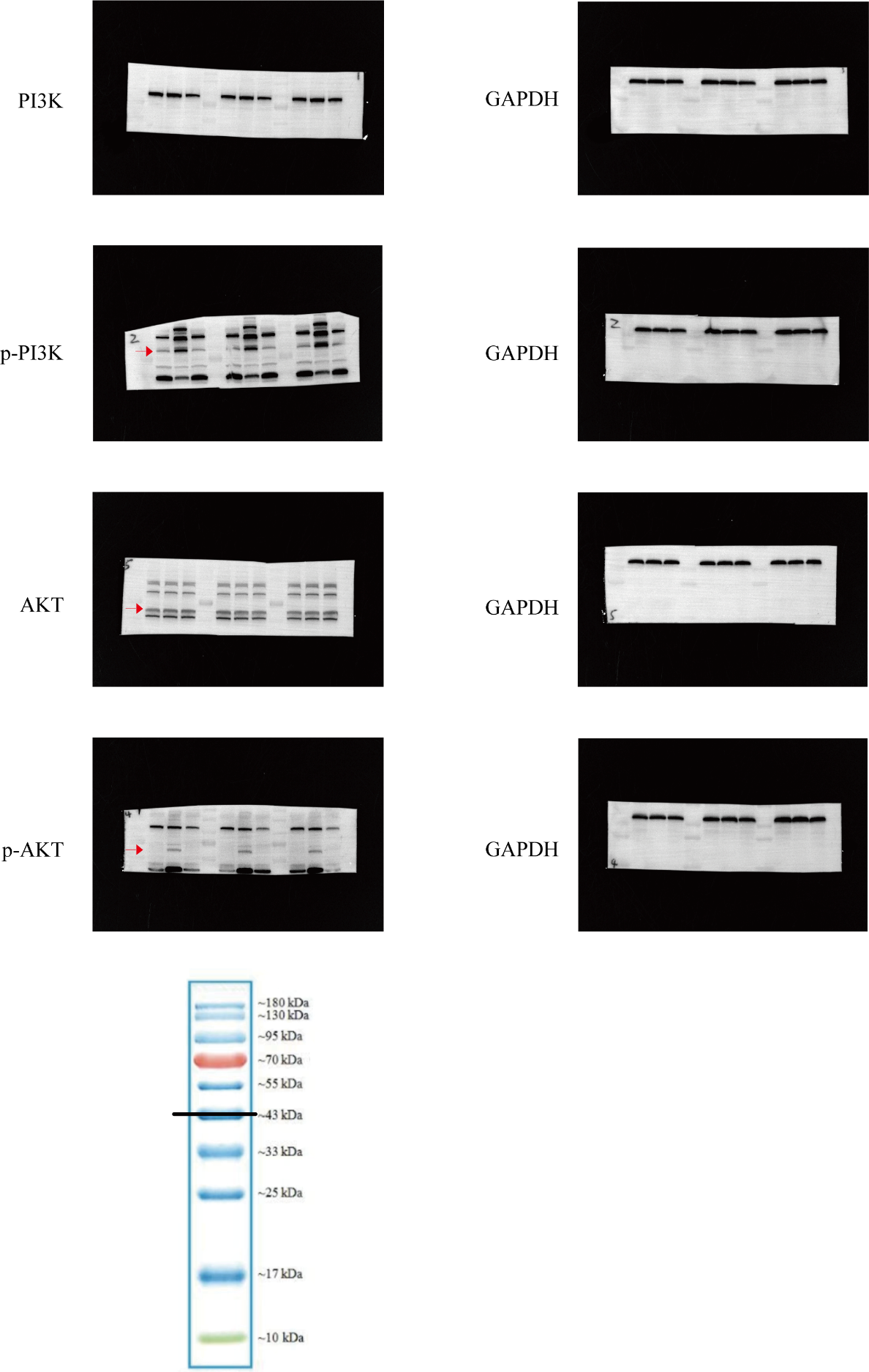


**Supplementary Figure 2.** Original Western blot images.
